# Supplementary material for: ESC: a comprehensive resource for SARS-CoV-2 immune escape variants
Source: Nucleic Acids Res. 2021 Oct 13;50(D1):D771–6. doi: 10.1093/nar/gkab895 (PMC8728294; doi:10.1093/nar/gkab895)
Supplement: gkab895_Supplemental_File [file gkab895_supplemental_file.pdf]

ESC - a comprehensive resource for SARS-CoV-2 immune escape variants

SUPPLEMENTARY DATASETS

Supplementary Figure 1. A brief comparison of the SARS-CoV-2 immune escape variant details from publicly available resources

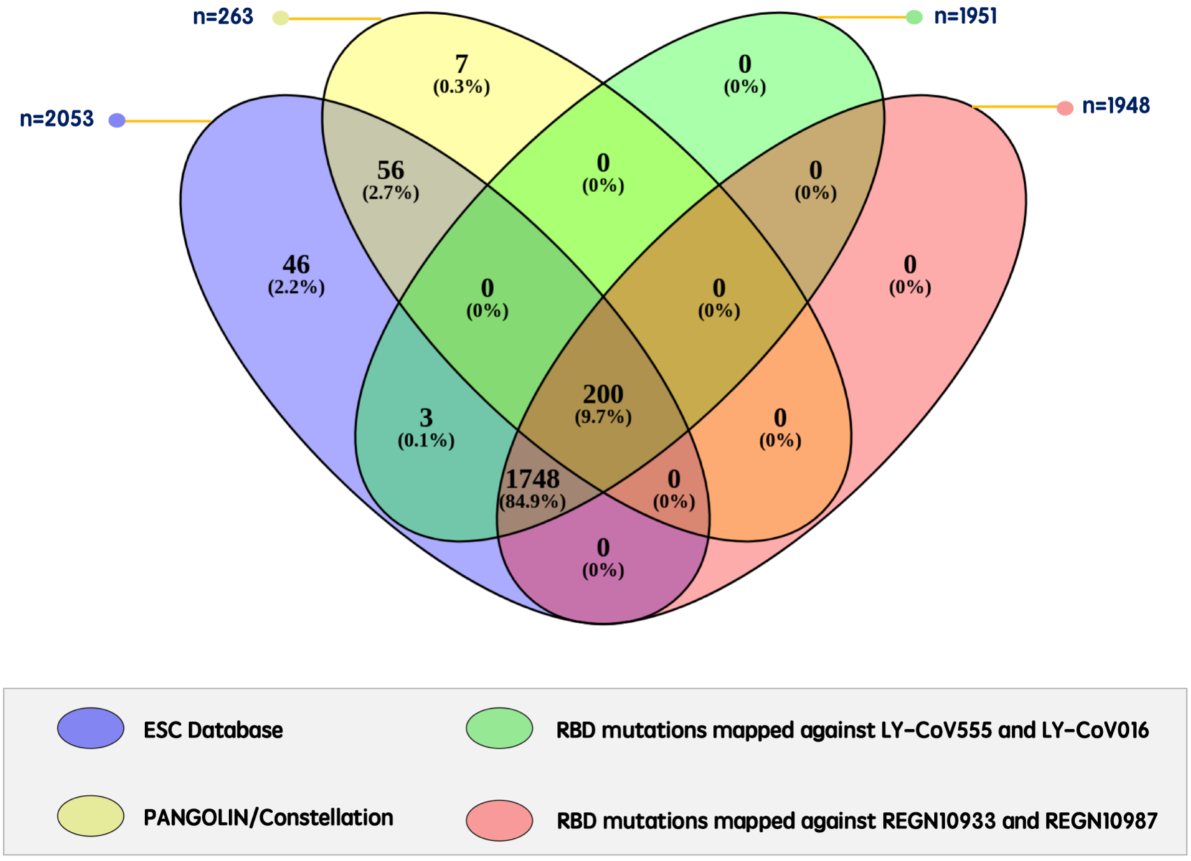

Supplementary Table 1a. Summary of publicly available resources with systematic collation of SARS-CoV-2 immune evasion variants

S.No	Resource/Repository	Contents and highlights	Total entries count	Resource/Repository Link
1.	PANGOLIN/Constellation	***List of immune escape variants*** in Spike protein along with the ***associated antibodies***	**263**	https://github.com/cov-lineages/constellations/blob/main/constellations/data/escape_list.csv (not available now)
2.	Complete map of SARS-CoV-2 RBD mutations that escape the monoclonal antibody LY-CoV555 and its cocktail with LY-CoV016	List of experimentally validated ***immune escape fractions*** for mutations sites in ***Spike RBD*** against ***LY-CoV555, LY-CoV016 and cocktail of LY-CoV555 and LY-CoV016***	**1951**	https://github.com/jbloomlab/SARS-CoV-2-RBD_MAP_LY-CoV555/blob/main/results/supp_data/LY_cocktail_raw_data.csv
3.	Prospective mapping of viral mutations that escape antibodies used to treat COVID-19	List of experimentally validated ***immune escape fractions*** for mutations sites in ***Spike RBD*** against ***REGN10933, REGN10987 and cocktail of REGN10933 and REGN10987***	**1948**	https://github.com/jbloomlab/SARS-CoV-2-RBD_MAP_clinical_Abs/blob/main/results/supp_data/REGN_and_LY-CoV016_raw_data.csv
4.	Coronavirus Antiviral and Resistance Database	List of variations and their corresponding escape fractions comprehensively collected from literature sources	**All reported VoCs and VoIs with isolated mutations also**	https://covdb.stanford.edu/page/susceptibility-data/

Supplementary Table 1b. A complete summary of the highlights and contents of the resources and repositories of SARS-CoV-2 immune escape variants

**Resource/Repository**	**Access**	**Interface options**	**Variant annotations**
PANGOLIN Constellation	Publicly available	No search interface	List of SARS-CoV-2 escape mutations with associated mAbs
RBD mutations mapped against LY-CoV555 and LY-CoV016	Publicly available	No search interface	Estimates of total and maximum escape fractions for RBD escape mutation sites against Ab cocktails
RBD mutations mapped against REGN10933 and REGN10987	Publicly available	No search interface	Estimates of total and maximum escape fractions for RBD escape mutation sites against Ab cocktails
Coronavirus Antiviral and Resistance Database	Publicly available	User friendly search options available	Provides fold reductions in neutralization against a range of monoclonal antibodies and patient sera
ESC database	Publicly available	User friendly search interface available	Annotations on<br>• VOCs/VoIs<br>• Protein Domains<br>• B cell and T cell Epitopes<br>• Associated mAbs,patient sera or vaccines<br>• Conservation sites<br>• Prediction of deleteriousness<br>• Sites spanning diagnostic primers/probes and potential error prone regions<br>• Global and geographical variant frequencies (including a graphical representation)<br><br>Extensive literature curation on<br>• Type of study<br>• Experimental details<br>• Details of neutralization escape efficacy

			- Generation of monoclonal antibodies and mutant viruses Other available linkouts - User friendly linkouts to GISAID and CoVDB have been provided for extensive search of the variants

Supplementary Table 2. List of custom annotation features used for ANNOVAR annotations

**Custom Datasets**
**Variation Type**
Non synonymous variant
Synonymous variant
**SIFT_Score**
**SIFT_Prediction (***Deleterious/Tolerable)*
**GERP**
**PhyloP**
**PhastCons**
**Uniprot_Domains**
**UNIPROT_Disulphite_Bond**
**UNIPROT_glyphos**
**UNIPROT_Transmembrane**
**IEDB_B-Cell_Epitopes**
**IEDB_CD4_Epitopes**
**IEDB_cd4Epitope_Score**
**IEDB_CD8_Epitopes**
**IEDB_cd8Epitope_Score**
**MPDI_Potential_Immunogenic_Regions**
**MPDI_Potential_Immunodominant_Epitopes**
**ARTIC_Primers**
**RT-PCR_Primers/Probes**
**Sequencing_Error_Sites**
**Homoplasic_Positions**
**Hypermutable_Sites**
